# A Rare Case of Histiocytic Sarcoma Secondary to Gastrointestinal Stromal Tumor in the Stomach: Transdifferentiation or Synchronicity?

**DOI:** 10.1155/2021/8856649

**Published:** 2021-03-08

**Authors:** Jiankun Tong, Jean Luo, Pierre F. Saldinger, William H. Rodgers

**Affiliations:** ^1^Department of Pathology, New York Presbyterian Queens, 56-45 Main Street, Flushing, NY 11355, USA; ^2^Department of Surgery, New York Presbyterian Queens, 56-45 Main Street, Flushing, NY 11355, USA; ^3^Weill Cornell Medical College, 525 East 68th Street, Box 130, New York, NY 10065, USA

## Abstract

Histiocytic sarcoma is a rare malignant histiocytic neoplasm composed of cells with morphologic and immunophenotypic features of mature tissue histiocytes. It occurs anywhere in the body and behaves aggressively. However, its etiology is unknown. Here, we report a 68-year-old female who developed histiocytic sarcoma following chemotherapy with imatinib (Gleevec) for gastrointestinal stromal tumor. Possible mechanisms of transdifferentiation from gastrointestinal stromal tumor to histiocytic sarcoma are discussed based on the features of our case and other two similar cases in the literature.

## 1. Case Presentation

A 68-year-old female was suspected to have recurrent gastrointestinal stromal tumor (GIST) of stomach and was admitted to the hospital for an exploratory laparotomy with gastric wedge resection of greater curvature.

**Figure 1:**
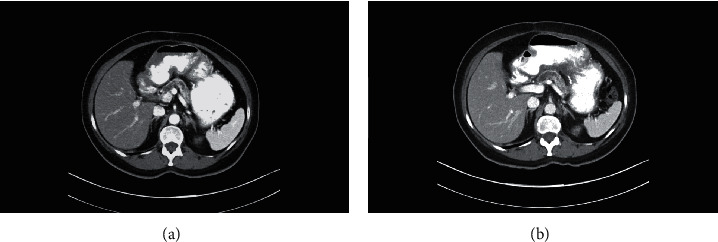
CT scan of abdomen showing a 13.1 × 11.4 × 10.7 cm mass from the posterior aspect of the gastric body (a). Repeat CT scan of abdomen revealing the persistent presence of the mass after chemotherapy with imatinib for 6 months (b).

**Figure 2:**
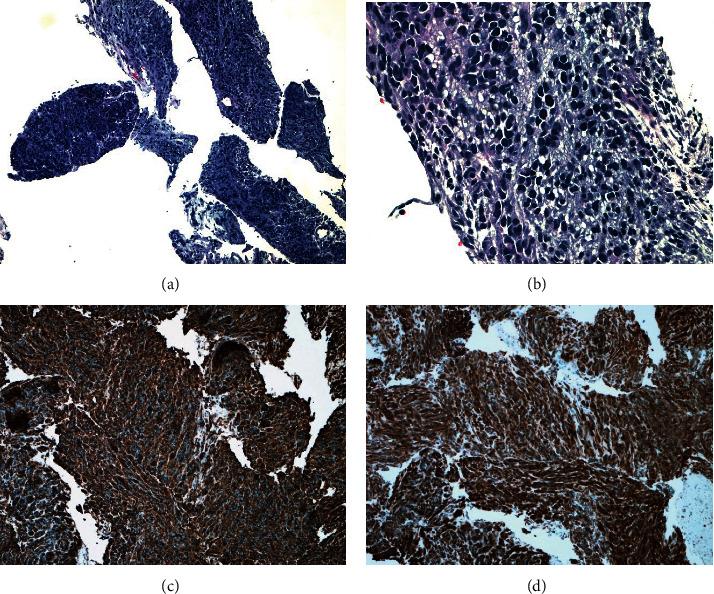
H&E sections showing atypical cellular proliferation of epithelioid cells and some spindle cells (×10) (a). The tumor cells showing round or oval nuclei with nuclear hyperchromasia (×40) (b) and expressing CD34 (c) and CD117 (d).

The patient had a history of GIST six years ago, being in remission after chemotherapy with imatinib (Gleevec) and partial gastrectomy. Four months ago, she had abdominal stress and chronic constipation. She denied fever, chills, nausea, vomiting, or abdominal pain. Laboratory analysis revealed mild anemia. An abdominal CT scan detected a gastric mass in the left upper quadrant, arising from the posterior aspect of the gastric body adjacent to the greater curvature and measuring 13.1 × 11.4 × 10.7 cm (Figure 1(a)). Endosonography confirmed a round intramural lesion in the body of the stomach, which appeared to originate from the muscularis propria involving the submucosal layer. Fine needle biopsy of the gastric mass showed epithelioid and short spindle cells with nuclear hyperchromasia and mild pleomorphism (Figures 2(a) and 2(b)). The tumor cells were positive for CD34 (Figure 2(c)) and CD117 (Figure 2(d))—immunohistochemical features that are diagnostic of gastrointestinal stromal tumor (GIST).

After treatment with imatinib for 6 months, the patient was admitted for selective partial gastrectomy after a follow-up CT scan of the tumor only indicated a slight interval decrease in size (Figure 1(b)).

## 2. Diagnosis

**Figure 3:**
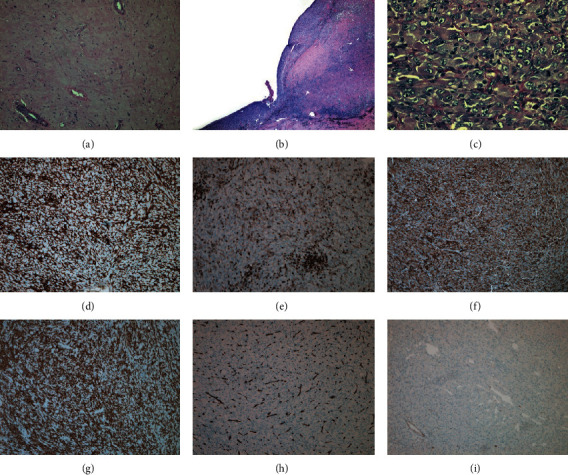
The 9.5 cm mass lesion consists predominantly of fibrotic stroma (80% of the mass) (×10) (a) with two distinct nodules measuring 1.0 cm and 0.6 cm (×2) (b). The two nodules are located in the inner surface of prior GIST  cavity showing large atypical epithelioid cells with abundant eosinophilic cytoplasm and prominent nucleoli (×40) (c). The cells were positive for CD4 (d), CD45 (e), CD68 (f), and CD163 (g), while negative for CD34 (h) and CD117 (i).

The partial gastrectomy specimen was submitted for histopathologic examination. Grossly, a mass lesion (9.5 cm) was identified between muscularis propria and serosa. The lesion consisted of a central cavity (6.0 cm) that contained two small distinct nodules (1.0 cm and 0.6 cm) along its border. The lesion consisted of fibrotic stroma (80% of the entire mass) with postchemotherapy effects (Figure 3(a)). This cavity represented the previous GIST biopsy site. Microscopically, both small nodules consisted of large atypical epithelioid cells with abundant eosinophilic, finely granular cytoplasm, and round nuclei with prominent nucleoli (Figures 3(b) and 3(c)). Mitotic figures were markedly increased (approximately 7 per 10 high power field). Small reactive lymphocytes were present in the background. Immunohistochemically, the large neoplastic cells were positive for CD4 (Figure 3(d)), CD10, CD31, CD45 (weak) (Figure 3(e)), CD68 (Figure 3(f)), CD163 (Figure 3(g)), HLA-DR, lysozyme, and vimentin, while they were negative for ALK-1, AE1/AE3, calretinin, CAM5.2, CD1a, CD3, CD15, CD20, CD21, CD30, CD34 (Figure 3(h)), CD35, CD43, CD117 (Figure 3(i)), CD123, chromogranin, desmin, DOG-1, EMA, HepPar-1, HMB-45, MART-1, MPO, S100, SMA, synaptophysin, TdT, and WT-1. The Ki-67 proliferative index was approximately 20–25%. The overall immunohistologic features were typical of histiocytic sarcoma (HS). Unexpectedly, there was no residual GIST despite histologic examination of the entire lesion. No BRAF mutation was detected by molecular analysis of both the original biopsy specimen of GIST and the current surgical specimen of HS.

## 3. Discussion

Histiocytic sarcoma (HS) is a rare malignancy that accounts for less than 1% of all hematolymphoid neoplasms [1, 2]. Lymph nodes are most commonly involved. The gastrointestinal tract is the most frequent extranodal site. Other less frequent extranodal sites are spleen, soft tissue, and skin [3, 4]. HS can be localized and disseminated. It affects the patients with a wide age range, from infancy to elderly; but it most commonly occurs in adults with the median age of 52 [3–6].

The rarity of HS makes it a difficult diagnosis, and it is often confused with other benign or malignant neoplasms. Its diagnosis is mainly based on morphology and immunohistochemistry, following exclusion of other hematopoietic and nonhematopoietic neoplasms. The former includes other histiocytic and dendritic cell neoplasms, myeloid sarcoma, anaplastic large cell lymphoma, and diffuse large B-cell lymphoma. The latter comprises malignant melanoma and undifferentiated or poorly differentiated carcinoma. Although each of the differential diagnoses shares morphologic and immunophenotypic features with HS, a correct diagnosis can be reached by careful examination and comprehensive immunohistochemical analysis.

The etiology of HS is unknown. Based on the 2017 World Health Organization (WHO) classification, HS is described as a malignant proliferation of cells showing morphological and immunophenotypic features of mature tissue histiocytes. Some cases report clonal IG and T-cell receptor gene rearrangements, most likely constituting examples of transdifferentiation [6]. Transdifferentiation, also known as lineage reprogramming, is the conversion of a cell type present in one tissue or organ into a cell type from another tissue or organ without going through a pluripotent cell intermediate. Previous publications report an association or concurrence of HS with a variety of malignancies, especially B-cell-associated hematopoietic neoplasms such as B-lymphoblast leukemia/lymphoma [7–10], chronic lymphocytic leukemia/small lymphocytic lymphoma [11], follicular lymphoma [12–15], hairy cell leukemia [16], MALT lymphoma [17], and mantle cell lymphoma [18]. It has been reported that HS occurred with myeloid neoplasms such as acute monocytic leukemia [19], chronic myeloid leukemia [20], chronic myelomonocytic leukemia [21], and idiopathic primary myelofibrosis [22]. These publications have provided direct and indirect evidence of the possibility of transdifferentiation between the two otherwise morphologically and immunohistochemically distinct neoplasms. A subset of HS cases occurs in patients with mediastinal germ cell tumors, most frequently malignant teratoma, with or without a yolk sac tumor component [23].

GIST is the most common mesenchymal tumor occurring in the gastrointestinal tract, especially in the stomach (56%), followed by the small intestine (32%), colorectum (6%), and esophagus (<1%) [24]. It rarely involves the omentum, mesentery, and peritoneum [24]. Interstitial cells of Cajal (ICCs) have been identified as the likely precursor cells of GIST. ICCs are located between the layers of the muscularis propria of the gastrointestinal tract in the regulation of gut peristalsis [25, 26]. They are thought to be pacemaker cells of the gastrointestinal tract. Immunohistochemically, they are usually positive for CD117 (KIT), CD34, and DOG-1. Some cases are focally or diffusely positive for SMA. Less than 5% of cases are weakly positive for Desmin and S100. Therefore, it is believed that these ICCs may have ultrastructural and immunophenotypic features of smooth muscle and neuronal differentiation.

In general, GISTs show a remarkable variability in their differentiation pathways, and GISTs can be roughly divided into four categories: tumors showing differentiation toward smooth muscle cells, neural-type elements, dual differentiation toward smooth muscle and neural-type elements, and those lacking differentiation toward either cell type [27]. Morphologically, GIST shows the spindle cell growth pattern, the epithelioid growth pattern, and the mixed growth pattern.

### 3.1. Is It Possible That HS in Our Case Is Transdifferentiated from GIST?

Recently, there have been two case reports of HS together with GIST. One report of HS in the intestine occurred in a previously treated case of GIST in the stomach, following two years of treatment [28]. A second case report documented synchronous HS and GIST as a single gastric mass [29]. Now, we report a rare case of a 68-year-old female developing HS secondary to GIST in the stomach after chemotherapy with imatinib and partial gastrectomy.

In our case, we observed two very interesting phenomena. First, both the GIST  and HS demonstrated a similar epithelioid morphology although the tumor cells exhibited different immunophenotypes and cellular sizes. The cell size of HS is much bigger than that of GIST. Secondly, the two nodules with HS are located in the inner surface of the mass cavity with stromal fibrosis, which represents the previous biopsy site of GIST. This may imply that the HS developed concurrently or subsequently from GIST after chemotherapy, suggesting that chemotherapy may be a triggering factor for the interstitial cells of Cajal (ICCs) or GIST to transdifferentiate to HS.

### 3.2. Can Treatment Cause a Transdifferentiation from GIST to HS?

It has been observed that the interval between the occurrence of lymphoma and that of HS varies between 2 months and 17 years [5]. In our case, the patient received imatinib treatment initially seven years ago and then again one year after recurrence of the mass. Imatinib therapy has been successfully used for treating chronic myeloid leukemia (CML). Imatinib discontinuation trials typically follow two distinct outcomes: one group with relapse and the other group remaining disease-free throughout the duration of follow-up [30]. Tang et al. explained this mechanism in the imatinib‐treated CML patients by suggesting that imatinib therapy leads to a rise of leukemic clones, which have different growth and differentiation properties compared to the predominant clone at the start of therapy [30].

Accordingly, transdifferentiation between cell types can occur naturally in response to tissue damage, and it can also be induced experimentally. It has been reported that blockade of KIT signaling induces transdifferentiation of interstitial cells of Cajal to a smooth muscle phenotype [31]. Perhaps transdifferentiation toward HS may be a product of the interaction of interstitial cells of Cajal with the effects of imatinib plus other intrinsic host factors, such as immune system and tumor microenvironment factors.

### 3.3. BRAF Mutation in Both GIST and HS

BRAF V600 E point mutation activates the preoncogene BRAF and has been reported in many hematopoietic and nonhematopoietic neoplasms. Although KIT and PDGFR are the two most common mutations in the GIST, BRAF V600 E mutation has been identified from 4% to 13% of the patients with KIT/PDGFRA wild-type GIST [32, 33]. BRAF V600 E mutation has also been found in some HS cases [16, 34, 35]. In one study, 5 of 8 HS cases were noted to have BRAF V600 E mutation [34]. To investigate whether BRAF V600 E mutation acts as a driver mutation favoring malignant transdifferentiation from GIST to HS, molecular studies for BRAF mutation were performed on previous and current specimens. No BRAF mutation was detected in either the GIST or HS tumors in our case.

### 3.4. RAS-MAPK Pathway Mutations

Just recently, Hornick's group has identified diverse and activating RAS-MAPK and PI3K-AKT-MTOR pathway mutations and CDKN2A tumor-suppressor gene alterations in the majority of histiocytic sarcomas by using a targeted next-generation sequencing approach [36]. Furthermore, a subset of cases harboring these mutations also show striking genetic similarities with B-cell lymphomas [36]. Whether these mutations concurrently exist in our GIST and HS cases is not clear. We believe that the targeted next-generation sequencing approach will provide a very useful tool to investigate these mutations and shed light on the future direction of demonstrating the possible mechanism of transdifferentiation from GIST to HS.

In conclusion, here, we reported a rare case of HS secondary to GIST in the stomach and discussed the possibility of transdifferentiation and synchronicity.

## Data Availability

The data used for the findings of this article are available and will be provided by the corresponding author upon request.
